# Noncoding RNAs in cancer therapy resistance and targeted drug development

**DOI:** 10.1186/s13045-019-0748-z

**Published:** 2019-06-07

**Authors:** Wen-Tao Wang, Cai Han, Yu-Meng Sun, Tian-Qi Chen, Yue-Qin Chen

**Affiliations:** 1 MOE Key Laboratory of Gene Function and Regulation, State Key Laboratory for Biocontrol, Sun Yat-sen University, Guangzhou, 510275 China; 2 School of Life Science, Sun Yat-sen University, Guangzhou, 510275 People’s Republic of China

**Keywords:** ncRNA, Chemoresistance, Radioresistance, Therapeutic approaches, Delivery strategies, Translational application

## Abstract

Noncoding RNAs (ncRNAs) represent a large segment of the human transcriptome and have been shown to play important roles in cellular physiology and disease pathogenesis. Increasing evidence on the functional roles of ncRNAs in cancer progression emphasizes the potential of ncRNAs for cancer treatment. Here, we summarize the roles of ncRNAs in disease relapse and resistance to current standard chemotherapy and radiotherapy; the current research progress on ncRNAs for clinical and/or potential translational applications, including the identification of ncRNAs as therapeutic targets; therapeutic approaches for ncRNA targeting; and ncRNA delivery strategies in potential clinical translation. Several ongoing clinical trials of novel RNA-based therapeutics were also emphasized. Finally, we discussed the perspectives and obstacles to different target combinations, delivery strategies, and system designs for ncRNA application. The next approved nucleic acid drug to treat cancer patients may realistically be on the horizon.

## Background

Cancer is an unconquered disease that generally causes mortality and morbidity worldwide and generates many adverse socioeconomic effects. Although protein-relevant therapeutics such as antibodies against Programmed Cell Death 1 (PD1), programmed death-ligand 1 (PDL1), and cytotoxic T-lymphocyte-associated protein 4 (CTLA-4) have driven a revolutionary trend in pharmacotherapy and drug development, some protein targets encoded by oncogenes are undruggable or inadequate for achieving remission, and cancer cells can acquire drug resistance [1]. Therefore, the treatment of cancer further requires multiple types of targets involved in oncogenic pathways for successful intervention.

**Fig. 1:**
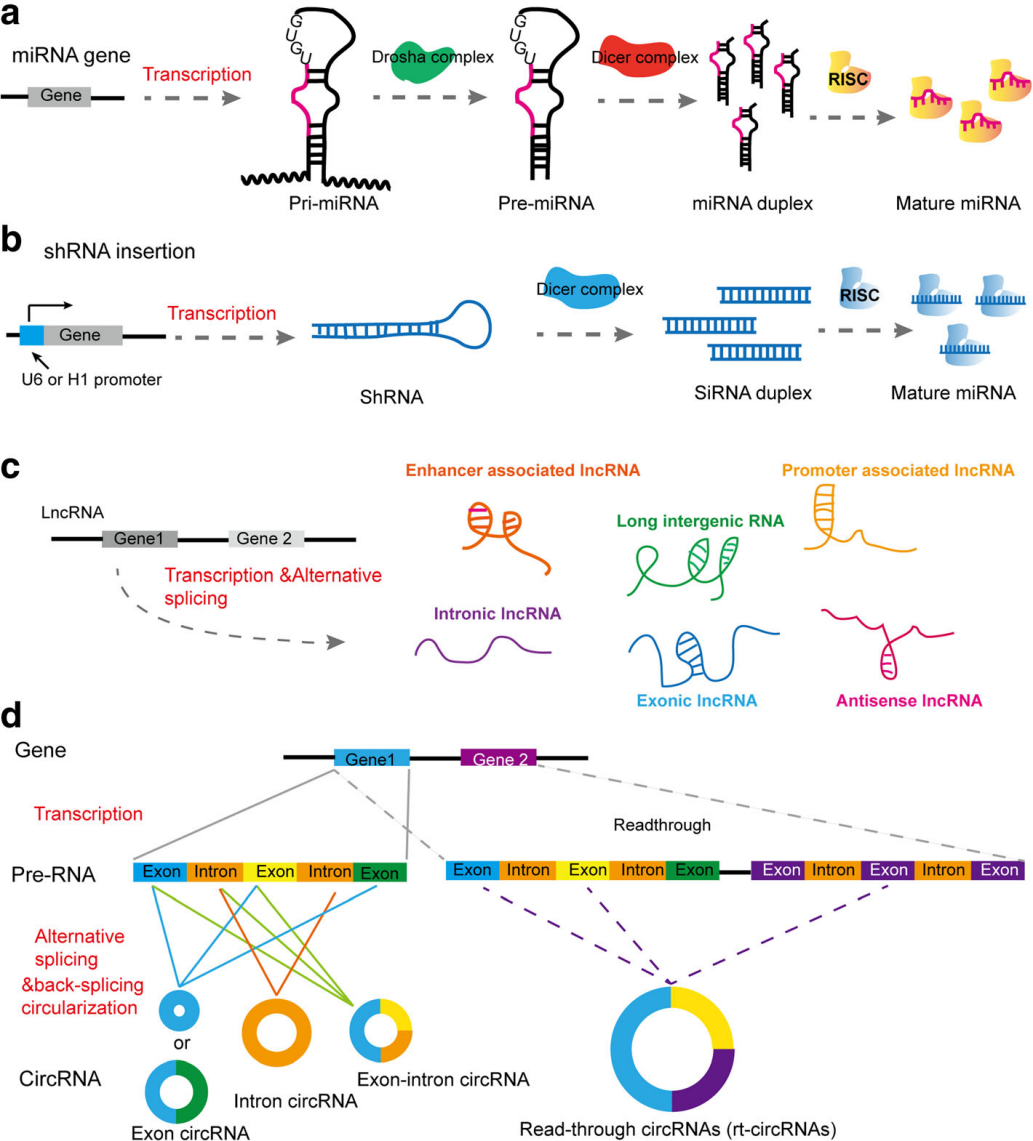
The biogenesis of several kinds of ncRNAs. **a** Most of miRNA genes are transcribed by Pol II and produce greater than 200-nt pre-miRNAs, which contain at least one hairpin structure harboring the miRNA sequence. In the nucleus, the pre-miRNAs are cleaved into approximately 70-nucleotide pre-miRNAs with a stem-loop structure by Drosha, an RNAse III enzyme. The pre-miRNAs are subsequently exported to the cytoplasm and then cleaved by another RNAse III enzyme, Dicer. Finally, the ~ 22 miRNA duplex was loaded into RISC and the mature single-stranded miRNA guides RISC to recognize mRNA targets. **b** The endogenous siRNA can be derived from shRNA. The transcription of shRNA gene is driven by a U6 or H1 promoter. ShRNA are then cleaved by Dicer to form mature ~ 21 siRNAs that subsequently are loaded into RISC. **c** LncRNAs are pervasively transcribed in the genome. According to the origins of transcription sites, lncRNAs can be summarized into different types, including enhancer-associated lncRNA, promoter-associated lncRNA, exonic and intronic lncRNA, long intergenic lncRNA, and antisense lncRNA. **d** Schematic representation of circRNA generation. Most of circRNAs are derived from pre-mRNAs and characteristic of spliceosome-dependent. CircRNA can be classified into various types, including exon circRNA, intron circRNA, and extron-intron circRNA. A novel type circRNA, called read-through circRNA (rt-circRNA), has been identified (marked in dotted line). The rt-circRNA is circularized from read-through transcripts

To advance the understanding of cancer initiation and progression, many genomic and proteomic approaches have been developed [1–3]. The landscape of genomic mutations in cancer reveals that many mutations or copy number changes in cancer are frequently located in noncoding DNA regions [1, 4]. Noncoding DNA has been reported to cover 95% of DNA sequences in the human genome, most of which are transcribed into tens of thousands of functional noncoding RNAs (ncRNAs), including microRNAs (miRNAs), small interfering RNAs (siRNAs), antisense RNAs (asRNAs), and long noncoding RNAs (lncRNAs) [4–7]. Recent studies have also reported a novel type of ncRNA, circular RNA (circRNA) [8–10]. A large portion of circRNAs are generated from exons of coding genes, and most do not express protein [8–10]. The biogenesis of several kinds of ncRNAs, such as miRNA [1, 2, 5], short hairpin RNA (shRNA) and siRNA [11], lncRNA [4], and circRNA [8–10], is summarized in Fig. 1. Accumulating evidence shows that ncRNAs are dysregulated and implicated in various cancer processes, such as cancer stem cell (CSC) initiation, metastasis, and drug resistance, highlighting the role of ncRNAs as potential therapeutic targets in cancer [5, 6, 8–15]. Several miRNAs have reached clinical trials [15–17]. In addition, lncRNAs and circRNAs have demonstrated significant clinical relevance in cancers due to their relatively complex and diverse structures and functions acting through multiple mechanisms [6, 8]. Moreover, the preclinical studies and increased success rates of nucleic acid therapeutics provide an opportunity to target ncRNAs for cancer treatment [5, 15–17].

Here, we summarize ncRNAs in therapeutic resistance, the potential as therapeutic targets, the current status of ongoing clinical trials, and therapeutic approaches for targeting ncRNAs. We also discuss the challenges for the efficient delivery of ncRNAs as therapeutics, the obstacles in clinical trials, and the perspective for the future design of nucleic acid therapeutics.

## NcRNAs in cancer therapy resistance

### NcRNAs in cancer chemoresistance

**Fig. 2:**
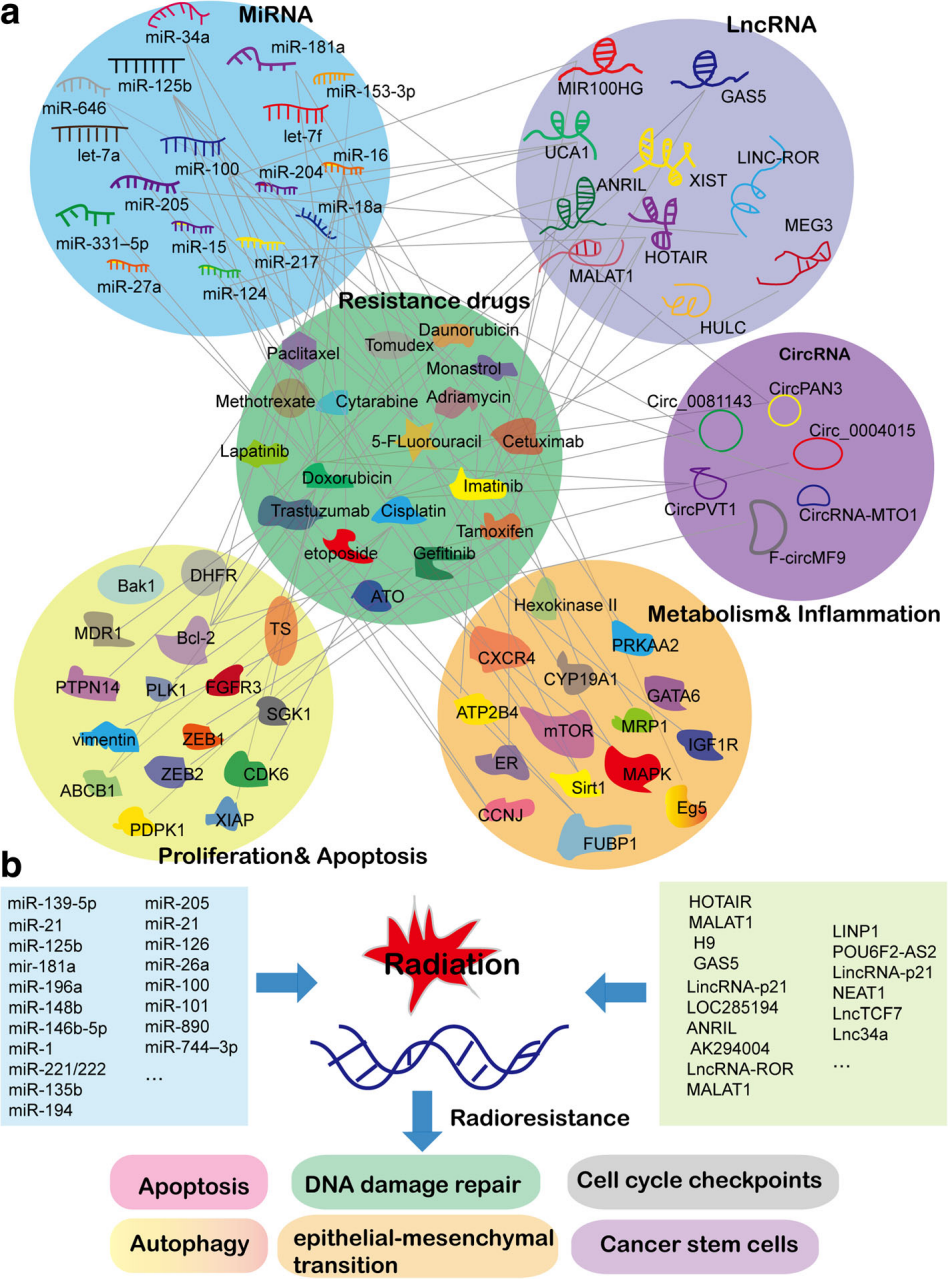
NcRNAs in cancer therapy resistance. **a** The network of miRNA, lncRNA, and circRNA in chemoresistance and the drug resistance pathways. **b** ncRNAs play a part in cancer radioresistance and concomitantly promote various events in the recurrence and metastasis of malignant tumors, including apoptosis, DNA damage repair, cell cycle checkpoints, autophagy, epithelial–mesenchymal transition, and cancer stem cells

The development of resistance to anticancer drugs is a major challenge in cancer therapy, generally causing relapse and even mortality in patients [18, 19]. Despite the complex mechanism underlying chemosensitivity and chemoresistance, ncRNAs are increasingly appreciated to overcome this obstacle. Figure 2a represents the known ncRNAs in cancer therapy resistance and the regulatory network of different kinds of ncRNAs involved in chemoresistance and the related drug resistance pathways.

#### MiRNA

MiRNAs are the most extensively studied ncRNAs in terms of chemoresistance or chemosensitivity [20], and some miRNAs exhibit double-faced roles in mediating the sensitivity of various tumors to different drugs [20–25]. An example is *miR-125*, which has been reported to resist drug therapy in various cancers [21, 22]. This miRNA confers paclitaxel resistance to breast cancer cells through suppressing the expression of the proapoptotic protein Bcl-2 antagonist killer 1 (*Bak1*) [22]; in addition, it can reduce the expression of dihydrofolate reductase (*DHFR*) and thymidylate synthase (*TS*) to promote the resistance of colon cancer and osteosarcoma to the drugs methotrexate or Tomudex [23]. However, the expression of *miR-125b* was also found to negatively correlate with 5-fluorouracil resistance in hepatocarcinoma [25]. The double-faced roles of miRNAs highlighted the necessity of thorough laboratory investigation of anti-miRNA drugs before proceeding to clinical trials [25].

*MiR-181a* is another example that mediates chemosensitivity. In patients with acute myeloid leukemia (AML) treated with similar intensive induction therapy regimens, a higher expression level of *miR-181a* was strongly correlated with complete remission (CR) [26]. Restoration of *miR-181a* levels by ectopic expression of artificial mimics reversed chemoresistance to cytarabine and daunorubicin in AML cell lines [27, 28]. Notably, lenalidomide, a drug in current clinical use, can induce the expression of miR-181a and therefore may be a possible therapeutic intervention for chemoresistant patients [29]. Other miRNAs, such as *let-7*, *miR-128*, *miR-331*, *miR-10*, and *miR-27a*, have also been shown to overcome chemoresistance in AML [30–33]. However, clinical datasets for these miRNAs are lacking or sometimes show contradictory results, necessitating larger cohort analyses. It should be noted that clinical correlation of ncRNAs with chemosensitivity does not necessarily guarantee the functional relevance of ncRNAs in drug resistance. Functional experiments assessing the effects of ncRNA modulation are essential.

#### LncRNA

LncRNA is another class of ncRNAs that has been linked to resistance to certain drugs in certain types of cancers [34, 35]. For example, blocking HOX transcript antisense RNA (*HOTAIR*) considerably decreased the expression of multidrug resistance-associated protein 1 (*MRP1*) and inactivated the PI3K/Akt signaling pathway, resulting in improved sensitivity to imatinib treatment [34, 35]. Another study reported that *HOTAIR* is upregulated in tamoxifen-resistant breast cancer tissues and that is restored upon the blockade of estrogen receptor (ER) signaling either by hormone deprivation or by tamoxifen treatment, leading to ligand-independent ER activity and tamoxifen resistance in breast cancer [36]. Thus, *HOTAIR* can be considered a potential therapeutic target for the reversal of resistance to conventional chemotherapeutics in patients with different types of cancer. In addition, Zhu et al. found that X-inactive specific transcript (*XIST*) regulated doxorubicin (DOX) resistance possibly through regulating the *miR-124*/*SGK1* axis and that *XIST* knockdown enhanced the antitumor effect of DOX in colorectal cancer (CRC) in vivo, providing insights into developing therapeutic strategies to overcome chemoresistance in CRC patients [37]. These findings show that lncRNAs are indeed closely related to chemoresistance, shedding new light on valuable therapeutic strategies against cancer.

#### CircRNA

The roles of circRNAs in chemoresistance have been demonstrated recently. For example, *hsa_circ_0001258* upregulated *GSTM2* expression through sponging *miR-744-3p* to promote chemoresistance in osteosarcoma [38]. *Hsa_circ_0081143* knockdown induced cisplatin sensitivity in gastric cancer cells in vitro and in in vivo mouse models through releasing *miR-646* to downregulate *CDK6* expression [39]. In addition, *hsa_circ_0004015* regulated the resistance of non-small cell lung cancer cells to tyrosine kinase inhibitors (TKIs) by targeting the *miR-1183/PDPK1* pathway [40]. Furthermore, overexpression of the fusion circRNA *f-circMF9* prominently increased the resistance of mouse *MLL-AF9* leukemia cells to arsenic trioxide (ATO) treatment in vivo [41]. Similar to lncRNAs, circRNAs may have opportunities to act as therapeutic targets for chemoresistance; however, additional investigation and testing are required.

## NcRNAs in cancer radioresistance

In addition to their roles in chemoresistance, ncRNAs play a part in radiosensitivity as summarized in Fig. 2b. As tumor cells generally impair DNA damage repair ability and have the propensity for more rapid division, these cells are more prone to apoptosis induced by radiation than normal tissues [42]. However, several findings have implied that radiotherapy might concomitantly promote the recurrence and metastasis of malignant tumors by activating epithelial–mesenchymal transition (EMT) and/or generating CSCs [43, 44]; importantly, ncRNAs are closely involved in these processes.

Several studies have revealed the strong relation between the expression patterns of a subgroup of miRNAs with the radiotherapy response in various cancers, including non-small cell lung cancer, head and neck tumors, squamous cell carcinoma, and squamous cervical carcinoma [45–48]. Thus, these miRNAs could be predictors of radioresistance (Fig. 2b, left panel). For example, Marina et al. found that several target genes of *miR-139-5p* were strongly predictive of outcome in radiotherapy-treated patients, suggesting that this miRNA may be a potentially useful predictive biomarker of radioresistance in breast cancer [46]. Besides, some miRNA expression levels change in a unique pattern during radiotherapy treatment. Thus, miRNAs can also be used as monitors to evaluate the real-time response to radiotherapy and to prevent delays in changing to an alternative treatment regimen [49–51]. The reported “circulating miRNAs” and plasma lncRNAs or lncRNAs such as *HOTAIR*, metastasis-associated lung adenocarcinoma transcript 1 (*MALAT1*), *H19*, and *GAS5* might also have value in the evaluation of radioresistance in cancer patients [52–55].

Mechanically, ncRNAs acting as modulators of radiosensitivity are mainly through regulating the processes of apoptosis, DNA damage repair, and EMT [52, 56]. For example, *miR-21*, *miR-125b*, *miR-181a*, *miR-196a*, and *miR-148b* suppress the expression of the apoptosis-related genes caspase-3, intercellular adhesion molecule-2 (*ICAM-2*), Protein Kinase C Delta (*PRKCD*), annexin A1 (*ANXA1*), or DNA methyltransferase 3b (*DNMT3B*) in a wide spectrum of cancers [56]. LncRNAs, such as *LincRNA-p21*, *LOC285194*, *ANRIL*, *AK294004*, *LncRNA-ROR*, and *MALAT1*, can regulate apoptosis-related genes either by binding to the protein partners or by acting as competing endogenous RNAs (ceRNAs) [57–59] (Fig. 2b, right panel). Some lncRNAs could also modulate radioresistance by regulating the DNA damage response [56, 60, 61]. For instance, lncRNA *LINP1* can enhance repair of DNA double-strand breaks by serving as a scaffold linking Ku80 and DNA-dependent protein kinase catalytic subunit (DNA-PKcs). Blocking *LINP1* increases the sensitivity of the tumor-cell response to radiotherapy in breast cancer [61]. Furthermore, many EMT- and CSC-related lncRNAs are also responsible for radioresistance [62–64]. *MALAT1* is the one reported to promote radioresistance through triggering EMT and CSC activity [63, 64]. Silencing the expression of *MALAT1* sensitizes nasopharyngeal carcinoma cells to radiotherapy both in vitro and in vivo through the inactivation of EMT and CSCs by modulating the *miR-1/SLUG* axis [64]. Some other lncRNAs, such as *LincRNA-p21*, *NEAT1*, *LncTCF7*, and *Lnc34a*, might also affect radiotherapy responses [65–67]. These findings highlight the effects of ncRNAs on CSCs and radioresistance, supporting the design of effective strategies to improve radiotherapy responses.

## NcRNAs as therapeutic targets

In addition to the involvement of ncRNAs in therapy resistances as discussed above, numerous ncRNAs have also shown potential as therapeutic targets because of their differential expression patterns between cancerous and normal tissues and their important roles in carcinogenesis [2, 5, 68–70]. With advances in nanotechnology, numerous clinical trials on RNA-guided precision medicine have recently been developed and/or are ongoing [2, 16, 71]. As summarized in Table 1, among the annotated ncRNAs, miRNAs are the most extensively investigated. MiRNAs function as either oncogenes or tumor suppressors, which, in turn, cause aberrant translational inhibition or degradation of their target mRNAs [72]. The pivotal double-faced roles of miRNAs in cancer make them as both therapeutic candidates and the direct therapeutic targets [17]. MiRNA mimics and anti-miRNAs are two major therapeutic forms used to mimic the function of endogenous tumor suppressor miRNAs and to deplete oncogenic miRNAs, respectively [73, 74]. For instance, *miR-34a* mimic was the first miRNA-based therapy to be introduced into the clinic [16]. *MiR-34a* can antagonize many different oncogenic processes by silencing oncogenes, including cyclin-dependent kinase 4/6 (*CDK4/6*), *SIRT1*, and *SOX2*, which function in self-renewal, migratory potential, and chemoresistance in various types of cancers [16, 75, 76]. In a phase I clinical trial, researchers designed a liposomal nanoparticle to deliver corresponding *miR-34a* mimics to the liver and precisely target cancer tissues [77]. More excitingly, many clinical trials of miRNA-based therapeutics have reached phase 3 or 4 ( https://clinicaltrials.gov), suggesting the promise of these therapeutic targets in precision medicine. Screenings of miRNA-based drugs have identified specific miRNA targets in different cancers, including *miR-31-3p* and *miR-31-5p* in colorectal cancer (CRC) [78], and *miR-2*1 and *miR-200* in oral cancer [79, 80]. The ongoing clinical trials have indicated that miRNA could be a widespread target in cancer treatment.

**Table 1:** Ongoing clinical trials of non-coding RNAs in cancer (ClinicalTrials.gov)

NcRNA	NCT number	Clinical trials	Cancer	Phases
miR-31 miR-210	NCT03684707	Cancer chemoprevention by metformin hydrochloride compared to placebo in oral potentially malignant lesions	Oral cancer	Phase 4
miR-31-3p miR-31-5p	NCT03362684	PETACC-8 miR-31-3p and miR-31-5p ancillary study	Colorectal Cancer	Phase 3
miR-34a	NCT02862145	Pharmacodynamics study of MRX34, microRNA liposomal injection in melanoma patients with biopsy accessible lesions	Melanoma	Phase 1 Phase 2
miR-34a	NCT01829971	A multicenter phase I study of MRX34, microRNA miR-RX34 liposomal injection	Primary liver cancer, SCLC, lymphoma, multiple myeloma, renal cell carcinoma	Phase 1
miR-10b	NCT01849952	Evaluating the expression levels of microRNA-10b in patients with gliomas	Glioma	Recruiting
miR-29b	NCT02009852	Observational study to explore the prognostic value of miR-29b in tissue, blood, and saliva	Oral cancer	Recruiting
miR-29 family	NCT01927354	Observational study to investigate the role of microRNA in Twist1-mediated cancer metastasis	Head and neck squamous cell carcinoma	Recruiting
miRNA-100	NCT02950207	Prospective observational study of antitumor activity correlation between hormonal therapy and expression miRNA100	Breast cancer	Recruiting
MiR-155	NCT03591367	The potential role of microRNA-155 and telomerase reverse transcriptase in diagnosis of non-muscle invasive bladder cancer and their pathological correlation	Bladder cancer	Recruiting
miR-16	NCT02369198	MesomiR 1: a phase I study of TargomiRs as 2nd or 3rd line treatment for patients with recurrent MPM and NSCLC	Malignant pleural mesothelioma, non-small cell lung cancer	Phase 1
miR-21 miR-20a-5 miR-103a-3p miR-106b-5p miR-143-5p miR-215	NCT02466113	A 6 microRNA tool for stratifying stage II colon cancer of receiving adjuvant chemotherapy	Colon cancer	Recruiting
miR-221 miR-222	NCT02928627	Clinical significance of hepatic and circulating microRNAs miR-221 and miR-222 in hepatocellular carcinoma	Hepatocellular carcinoma	Recruiting
miR-122	NCT03687229	The effect of DAAs on miRNA-122 and insulin resistance in chronic HCV patients	Chronic hepatitis C, hepatocellular carcinoma	Not yet recruiting
MiR-25	NCT03432624	Detection of microRNA-25 in the diagnosis of pancreatic cancer	Carcinoma, pancreatic ductal	Not yet recruiting
HOTAIR	NCT03469544	Long non-coding RNA HOTAIR and Midkine as biomarkers in thyroid cancer	Thyroid cancer	Recruiting
THRIL,PACER	NCT03057171	A study on the gastrointestinal disease and helicobacter pylori controlled long non-coding RNA	Stomach cancer	Recruiting

Apart from the extensively studied miRNAs, lncRNAs and circRNAs have recently emerged as novel targets [6, 8, 9, 68]. Compared with miRNAs, lncRNAs and circRNAs act through more diverse mechanisms in carcinogenesis [4, 81, 82]; thus, targeting lncRNAs and circRNAs provides varied means to modulate a range of critical processes in cancer development. Double-stranded RNA-mediated interference (RNAi) and single-stranded antisense oligonucleotides (ASOs) are two major approaches to target lncRNAs. ASOs can reduce the levels of oncogenic isoforms of lncRNAs by regulating alternative splicing, modulating RNA–protein interactions, or causing lncRNA degradation [83, 84]. For example, targeting lncRNA *MALAT1* with ASO induced differentiation and inhibited metastasis in a mouse model of breast cancer [84, 85]. The antimetastatic effect of *MALAT1* targeting by ASO was also reported in a lung cancer xenograft model, highlighting the potential of *MALAT1* as a therapeutic target in multiple tumors [86]. Notably, a subset of lncRNAs named natural antisense RNAs (NATs) are sometimes located near important tumor suppressors, such as *ANRIL* and *p21-AS* [87, 88]. Therapeutic inhibition of *cis*-acting NATs with a special type of ASO, antagoNATs, can potentially upregulate the overlapping tumor suppressor genes; thus, modulating lncRNA expression could be a tool to regulate gene expression. However, therapeutic targeting of lncRNAs and circRNAs remains mainly at the laboratory stage.

## Therapeutic approaches for targeting ncRNAs in cancers

**Fig. 3:**
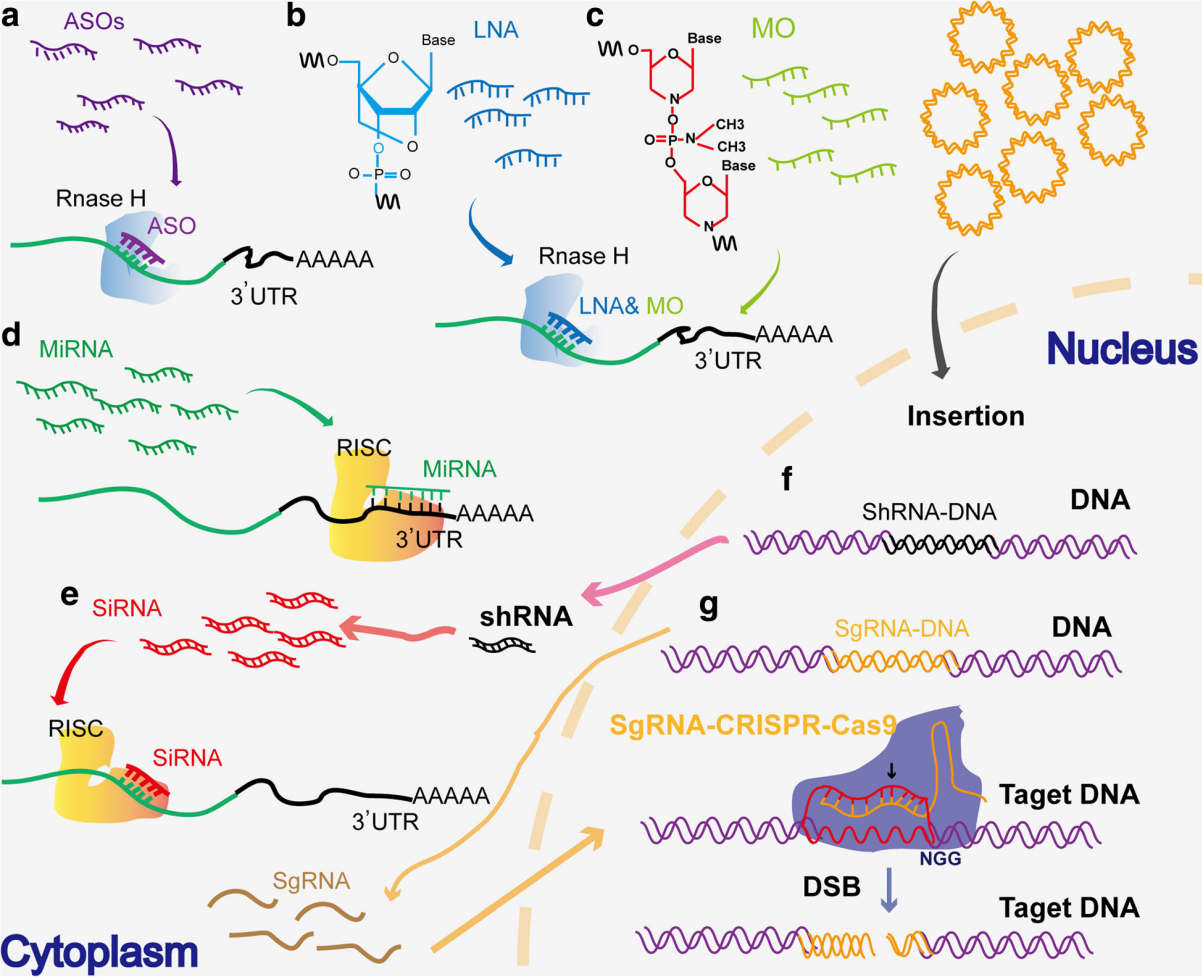
Schematic illustration of ncRNAs in cancer therapy from delivery to targeting. **a** ASO sequence searching and hybridization to the cognate site of mRNA and RNase H1 recruitment and cleavage. The schematic illustration of LNA (**b**) and MO (**c**) molecules, and their sequence hybridization to the cognate site of mRNA and RNase H1 recruitment and cleavage. **d** The mature miRNAs incorporated into RISC, then binded with a 6mer to 8mer seed sequence to the 3′UTR of an mRNA molecule, complementarity targeting the mRNA transcript for degradation, and imperfect complementarity inhibiting translation. **e** SiRNA interacts with RISC and binds to the target mRNA, resulting in the mRNA degradation. **f** Selective infectivity of the oncolytic virus shows that the delivery vehicle armed shRNA into cancer cells and inserted into DNA. The system can restrict shRNA expression to the cancer microenvironment and is expected to augment antitumor outcomes by siRNA-mediated knockdown of oncogene expression. **g** Engineering of 20 nucleotides in the sgRNA can be specifically delivered and expressed in cancer cells. The expressed sgRNA combines with Cas9 can recognize the complementary DNA sequence and generate the site-specific genomic double-strand breaks (DSBs)

Approaches for therapeutic targeting are essential for precision medicine. Several preclinical studies have been initiated to investigate anticancer strategies for targeting oncogenic ncRNAs (https://clinicaltrials.gov). Three strategies have been proposed: ASOs, locked nucleic acids (LNAs), and morpholinos [1, 89–94]. ASOs are single-stranded oligonucleotides that have specific complementarity to target sequences to promote target RNA degradation by RNase H as shown in Fig. 3(a) [91]. LNAs are also single-stranded oligonucleotides containing a stretch of DNA flanked by LNA nucleotides and offer specific complementarity and RNase H-mediated degradation of the target sequence as shown in Fig. 3(b) [92, 93]. Different from ASOs and LNAs, morpholino oligonucleotides (MO) are 25-nt nonionic DNA analogs used to promote RNA degradation through binding target RNAs in diverse organisms (Fig. 3(c)) [94–97]. These strategies have been applied for targeting oncogenic ncRNAs in cancer. For instances, *miR-10b* ASOs together with a low dose of doxorubicin showed a significant decrease in tumor size compared with the results only using doxorubicin monotherapy to treat breast cancer in mouse models [98]. The researchers also applied *miR-10b* LNAs for investigation and found that *miR-10b* LNAs can enhance the sensitivity of breast cancer to doxorubicin in mouse models, with no additional damage to normal tissue, suggesting low toxicity associated with the delivery of this LNA nanoparticle [98]. *MALAT1* ASOs could also inhibit the metastasis of cancer cells and the tumor burden in mice [99]. AVI-4126, a morpholinos-based drug, was used to inhibit *c-MYC* translation in a sequence-specific manner by simultaneously blocking the expressions of *c-MYC* and causing the mis-splicing of its pre-mRNA, resulting in significant growth inhibition in various cancer cells, such as prostate cancer, breast cancer, and lung cancer [95, 97, 100]. Chang et al. designed a phosphorodiamidate morpholino oligomer that effectively silenced *miR-487a* in a mouse model and reduced tumor growth and metastasis [96]. These studies indicate that morpholinos-based drugs of targeting oncogenic ncRNAs may represent a promising approach for cancer therapy. Further clinical trials are required.

## Small ncRNAs as a therapeutic tool in cancer treatment

With unique properties in their chemical behavior, modes of action and clinical pharmacokinetics, ncRNAs have been considered not only as the therapeutic targets, but also as an alternative promising therapeutic tool for cancer treatment. This possibility is especially meaningful for small ncRNAs, including miRNAs (Fig. 3(d)) [2], siRNAs, shRNA (Fig. 3(e, f)) [101], small activating RNAs (saRNAs) [7], guide RNAs (gRNAs) (Fig. 3(g)) [102], and other forms of small RNAs (sRNAs) [1, 6].

SiRNA is a well-studied small ncRNA tested for preclinical trials. SiRNAs are a class of synthetic short double-stranded RNAs with 21 base pairs that are recruited by the RNA-induced silencing complex (RISC) in cells and silence the expression of their target mRNAs, lncRNAs, or circRNAs by complementary base pairing (Fig. 3(e)) [101, 103]. Zorde et al. developed a system for local prolonged effects of siRNA (Local Drug EluteR, LODER) enabling the utilization of siRNAs against mutated Kirsten rat sarcoma (*KRAS*) (siG12D LODER) [104]. Mutated *KRAS* has been reported to be resistant to small molecule drugs that target oncogenic proteins. They found that siG12D LODER suppressed the growth of human pancreatic cancer cells in vivo and therefore prolonged mouse survival [104]. In an open-label phase 1/2a study, an improved therapeutic effect was reported in patients with locally advanced pancreatic cancer (LAPC) treated with a combination regimen of siG12D-LODER and gemcitabine [105]. Some other siRNA-based drugs have already completed phase II clinical trials as shown in Table 2, including DCR-MYC for *MYC* knocking down to arrest cell growth in hepatocellular carcinoma (HCC) (NCT02314052), Atu027 for *PKN3* knocking down to regulate cell migration in metastatic pancreatic adenocarcinoma (NCT01808638), etc. The rapid development of siRNA- and miRNA-based clinical trials profits from the shortness of small RNA sequences and technical advances in previously introduced delivery carriers. These advantages facilitate enduring and safe circulation in the blood and ease of uptake by target cells to improve the biodistribution and bioavailability of these RNAs during trafficking to cancer cells.

**Table 2:** Ongoing clinical trials with identified siRNAs in cancer (ClinicalTrials.gov.)

SiRNA targets	NCT number	Title	Cancer	Phases
VEGF	NCT00306904	Safety and efficacy study of small interfering RNA molecule (Cand5) to treat diabetic macular edema	Diabetic macular edema	Phase 2
MYC	NCT02314052	Phase Ib/2, multicenter, dose escalation study of DCR-MYC in patients with hepatocellular carcinoma	Hepatocellular carcinoma	Phase 1 Phase 2
PD-L1 PD-L2	NCT02528682	MiHA-loaded PD-L-silenced DC vaccination after allogeneic SCT	Hematological malignancies	Phase 1 Phase 2
Mutated KRAS	NCT01676259	A phase 2 study of siG12D LODER in combination with chemotherapy in patients with locally advanced pancreatic cancer	Pancreatic ductal adenocarcinoma pancreatic cancer	Phase 2
PKN3	NCT01808638	Atu027 plus gemcitabine in advanced or metastatic pancreatic cancer (Atu027-I-02)	Carcinoma, pancreatic ductal	Phase 1 Phase 2
EphA2	NCT01591356	EphA2 gene targeting using neutral liposomal small interfering RNA delivery	Advanced cancers	Phase 1
LMP2 LMP7 MECL1	NCT00672542	Immunotherapy of melanoma with tumor antigen RNA and small inhibitory RNA transfected autologous dendritic cells	Metastatic melanoma Absence of CNS metastases	Phase 1
APN401	NCT02166255	APN401 in treating patients with melanoma, kidney cancer, pancreatic cancer, or other solid tumors that are metastatic or cannot be removed by surgery	Recurrent melanoma Recurrent pancreatic cancer Recurrent renal cell cancer Stage III/IV pancreatic cancer Stage III renal cell cancer Stage IIIA/B/C/IV Melanoma Stage IV renal cell cancer	Phase 1
PLK1	NCT01437007	TKM 080301 for primary or secondary liver cancer	Colorectal cancer with hepatic metastases Pancreas cancer with hepatic metastases Gastric cancer with hepatic metastases Breast cancer with hepatic metastases Ovarian cancer with hepatic metastases	Phase 1
M2	NCT00689065	Safety study of CALAA-01 to treat solid tumor cancers	Solid tumor	Phase 1
AHR	NCT01075360	The role of aromatic hydrocarbon receptor in the tumorigenesis of neuroblastoma and its relationship with MYCN expression	Neuroblastoma	Recruiting
B4GALNT3	NCT01058798	The role of glycosyltransferases in the oncogenesis of neuroblastoma	Neuroblastoma	Recruiting
BCL-B	NCT01270009	Role of BCL-B in multiple myeloma	Multiple myeloma	Not yet recruiting

saRNA is another type of small double-stranded ncRNA designed to target gene promoters to activate transcription and thus upregulate gene expression [7]. In a preclinical trial, Reebye et al. designed a saRNA targeting *CEBPA* that can activate the transcription of *C/EBP-α* in a liver cancer model [106]. They found that intravenous injection of *C/EBPα*-saRNA reduced the tumor burden and suppressed the expression of interleukin (IL) 6R and c-Myc and inhibited STAT3 phosphorylation [106]. The delivery efficiency was improved by loading *C/EBPα*-saRNA in a liposomal nanoparticle in the following clinical trial study [107]. The ongoing clinical trial indicated that saRNAs are promising for activating the transcription of tumor suppressor.

gRNA is also an important type of small ncRNAs. Since the discovery of the clustered regularly interspaced short palindromic repeats (CRISPR)–CRISPR-associated (Cas) system, Cas-gRNA-based biotechnology has developed rapidly and massively (Fig. 3(g)) [102, 108]. Diverse RNA-programmable CRISPR–Cas enzymes have been gradually found and subsequently applied to reverse the aberrant expression of oncogenes and tumor suppressor genes [102]. Generally, the CRISPR–Cas system has two advantages over other gene editing strategies. First, the CRISPR–Cas system offers sequence-specific DNA targeting through a single-guide RNA (sgRNA)-based nucleoprotein complex that specifically cleaves the genomic DNA of interest to accomplish gene editing and mutation. Second, designing the sgRNA sequence targeting the desired DNA sequence is simple and flexible. Due to the great advantages of the sgRNA-guided CRISPR–Cas system, CRISPR systems have been widely adapted to facilitate the discovery of new targets in cancer therapy. For example, Yamauchi et al. performed a genome-wide CRISPR–Cas9 screen in AML cell lines to identify novel targets for AML therapy and discovered that the mRNA decapping enzyme scavenger (*DCPS*) gene, which is involved in pre-mRNA metabolic pathways, is essential for AML cell survival [109]. More interestingly, these researchers further found that germline biallelic *DCPS* loss-of-function mutations resulted in failure to induce leukemogenesis in humans, suggesting that CRISPR–Cas9-mediated silencing of *DCPS* is a potential strategy for AML therapy [109]. In addition, recent applications of the CRISPR–Cas system in chimeric antigen receptor (CAR) T cells, including CD133-specific CAR T cells with PD-1 deficiency and CD3-specific CAR T cells with diacylglycerol kinase (DGK) deficiency [110, 111], have been shown to be promising strategies in cancer immunotherapy. Although most studies exploring the CRISPR–Cas system are still in the preclinical stage, multiple Cas-based clinical trials are in progress or will be commencing soon.

## NcRNA delivery strategies for potential translational application

**Fig. 4:**
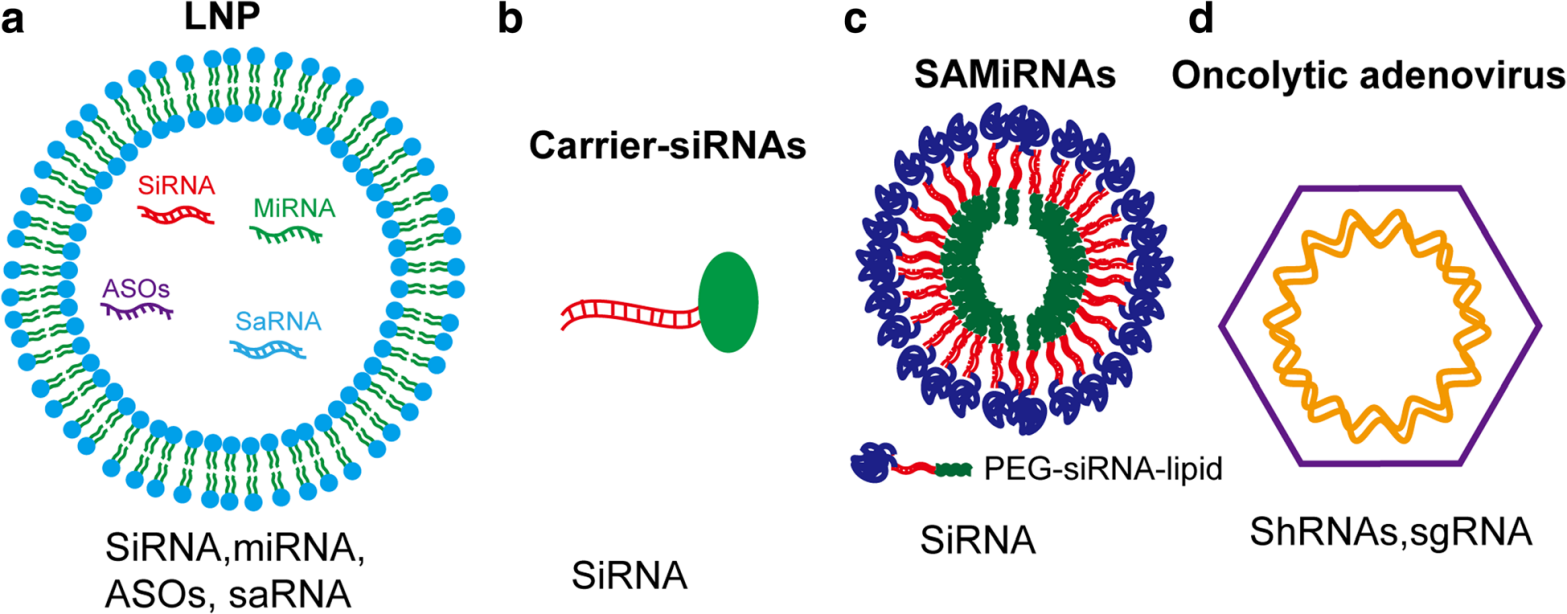
Schematic illustration of the delivery strategies of ncRNAs in cancer therapy. **a** SiRNA, ASOs, saRNA, and miRNA can be encapsulated inside the LNP to be protected from biological conditions and delivered into cancer cells. **b** SiRNA is chemically conjugated with carriers forming carrier-siRNA conjugates. **c** SAMiRNA, the siRNAs are modified with lipid and PEG molecules, and then self-assembled lipid nanoparticles. **d** ShRNA and sgRNA can be delivered by oncolytic adenovirus-mediated strategy and achieve a long-lasting expression of ncRNA in cancer cells.

Although small ncRNAs have been shown to be promising and effective therapeutic drugs in vitro, the low bioavailability of these nucleic acid drugs in vivo is a major challenge [6, 73, 94]. Thus, the development of advanced drug delivery strategies is urgently needed. To overcome the general problems of a short half-life, off-target effects and low transfection efficiency in RNA delivery, various small ncRNA carriers or systems have been proposed and extensively investigated, including nanoparticles (Fig. 4a), ncRNA modification (Fig. 4b, c), and oncolytic adenovirus strategy (Fig. 4d).

The first class of carriers is nanoparticle-based and includes self-assembled oligonucleotide nanoparticles [112], lipid-based nanoparticles (LNPs) [113], inorganic nanoparticles, and polymeric nanoparticles [101]. Huang et al. reported the assembly of a calcium–phosphate–lipid nanoparticle, which showed higher efficiency in delivering siRNA into HCC cells than into normal hepatocytes [114]. These lipid nanoparticles were subsequently loaded with VEGF siRNAs and downregulated VEGF expression specifically in HCC both in vitro and in vivo, exhibiting a potent antiangiogenic effect in the tumor microenvironment in a mouse model of HCC [115]. This carrier spectrum is also reflected in the miRNA monotherapies used in several types of cancers—for example, *miR-34* and *miR-125b* in non-small cell lung cancer [116, 117], *miR-212* and *miR-216b* in pancreatic ductal adenocarcinoma [118, 119], *miR-328* in CRC [120], and *miR-221* in liver cancer [121]. Several clinical trials for siRNA-/miRNA-based therapeutics targeting cancers have been performed (Fig. 3(d, e)).

NcRNA modification is another effective strategy to improve the precision and durability of small ncRNAs in targeting genes of interest in cancer, including siRNAs chemically conjugated with carriers forming carrier-siRNA conjugates (Fig. 4b) and siRNAs modified with lipid and PEG molecules, and then self-assembled lipid nanoparticles named as SAMiRNA (Fig. 4c). In developing therapy for liver cancer, two cooperative groups have chemically conjugated special siRNAs with (2–3)*N*-acetylgalactosamine (GalNAc) and developed GalNac-conjugated siRNAs [122]. GalNAc is a type of ligand with high binding affinity to the asialoglycoprotein receptor (ASGPR), which is specifically expressed and localized on the surface of hepatocytes. Martin et al. demonstrated that the interaction of ASGPR with the GalNAc ligand facilitates clathrin-mediated endocytosis [123]. Finally, most GalNac-conjugated siRNAs could be precisely delivered into the lesions of HCC and form RISC complexes to degrade target mRNAs [122]. Currently, the developed GalNAc–siRNA conjugates are undergoing clinical trials. The team subsequently refined this siRNA by optimizing the positioning of the 2'-deoxy-2'-fluoro and 2'-O-methyl ribosugar modifications to enhance stability without compromising the intrinsic RNAi activity, yielding significantly improved potency and duration in preclinical species [124]. Another challenge is the cytotoxicity induced by delivery carriers such as cationic liposomes, which can trigger pulmonary inflammation and the production of reactive oxygen intermediates [125]. For instance, siRNAs conjugated to the cell-penetrating peptide (CPP) TAT(48-60) indeed exhibited improved gene silencing efficiency. However, this conjugate also concomitantly activates the innate immune response [126]. Modification of siRNAs with hypotoxicity is also a problem requiring a solution. Yoon et al. developed SAMiRNAs by conjugating siRNAs to hydrophilic polymers and lipids to form conjugates (Figs. 4c and 3 (e)) [112]. SAMiRNAs can be specifically delivered into tumor tissue with high stability and have high knockdown efficiency. The advantage of this method is that the induction of cytokines in mouse peripheral blood mononuclear cells (PBMCs) and the levels of toxicity in liver and kidneys were below the relevant detection limits [112].

The methods described above are based on transient transfection of dsRNA. However, effective siRNA targeting for therapeutic purposes would require sustaining effects of gene silencing on oncogenic RNAs of interest. The emergence of a stable RNA silencing strategy via oncolytic adenovirus has introduced a revolutionary advance in cancer therapy [127]. The oncolytic adenoviral vector is designed to remove the partial sequences of the *E1A* gene region to render the adenovirus replicative exclusively in cancer cells [127]. Thus, compared to conventional therapy, this engineered adenovirus showed reduced toxicity and achieved promising results (Figs. 4d and 3(f, g)) [127–130] . The oncolytic adenoviral vector allows the insertion of large numbers of different genes with various DNA sequence lengths, including human genes, shRNA sequences, and CRISPR–Cas9 sequences. We consider Ad-shRNA as an example. Machitani et al. developed a telomerase-specific replication-competent adenovirus (TRAD), which carries the tumor-specific promoter-driven *E1* gene expression cassette and exhibits increased replication efficiency and antitumor activity [130]. This group loaded shRNA targeting Dicer into the TRAD. After infection, TRAD-shDicer efficiently induced Dicer knockdown and exhibited significantly higher replication efficiency and prodeath activity in tumor cells than in normal cells [130]. Continued progress in the development of oncolytic adenovirus strategies might allow this approach to be an important and powerful alternative tool to treat cancer.

## Conclusions and perspectives

NcRNAs are emerging as crucial players in tumorigenesis. Recent progress in biotechnologies such as high-throughput sequencing, genome editing, mouse modeling, and pharmaceutical chemistry has allowed functional studies of ncRNAs to provide a new perspective for waging the war against cancer. In addition to miRNAs and lncRNAs, other novel ncRNAs, such as transfer RNA (tRNA) fragments, snoRNA-related lncRNAs (sno-lncRNAs), and circRNAs, have also begun to appear on the radar of cancer researchers. Notably, snoRNAs have regained attention in cancer research, and snoRNA derivatives might be potential players in cancer development. The tissue-specific expression of ncRNAs makes them exciting candidates for molecular targeting. Additional ncRNA targets for cancer treatment are expected to be discovered in subsequent studies. However, because ncRNAs vary in length and modes of action, the development of systematic genomic and functional approaches will be needed to better understand the roles of ncRNAs and to evaluate their potential as therapeutic targets.

As chemotherapy and radiotherapy remain the mainstream treatment approaches for cancer patients, the roles of ncRNAs in mediating chemo- and radioresistance will be increasingly appreciated. Although a subgroup of ncRNAs—to date, miRNAs and lncRNAs, in particular—have proven to be useful biomarkers for predicting treatment outcomes or monitoring therapeutic responses, most studies are still in the preclinical stage. In addition, only a few of these ncRNAs exist stably in body fluid, thus enabling a noninvasive liquid biopsy approach. More efforts are needed to discover additional circulating ncRNAs for convenient clinical diagnosis. Notably, the results of some studies evaluating the potential of ncRNAs as biomarkers are conflicting. Larger cohorts of clinical data should be mined to reconcile these controversies.

Not only are ncRNAs promising targets for treating cancer and modulating cancer treatment sensitivity, approaches for targeting ncRNAs could be RNA-based. The rapid evolution of nucleic acid therapeutics offers an exceptional opportunity to explore ncRNAs as druggable targets in the clinic. MiRNA mimics and modified miRNAs/siRNAs are currently major RNA-based drugs that target mRNAs and ncRNAs. Other methods, such as ASOs, morpholinos, and small molecules, are also promising approaches via the modulation of ncRNA degradation, alternative splicing, and RNA–protein interactions. The CRISPR–CAS system represents another prospective method, but much additional study is needed for its eventual application in the clinic. Despite the enthusiasm, several obstacles still need to be overcome. First, delivery technologies with increased efficiency should be developed; crossing the cell membrane remains the foremost issue. The complex internal microenvironment makes the delivery and application of ncRNA difficult, including RNA degradation and instability, off-target, and low transfection efficiency. Development of more advanced delivery strategy is urgency. The combination of two or more carriers may be a good choice for ncRNA targeting, such as combination of nanodesigns with organ-specific response receptor may improve the precision and efficiency of drug delivery. Second, identifying a means to evade nuclease degradation or innate immune system targeting is critical for increasing bioavailability. Last but not least, minimal off-target effects and toxicity should be ensured. Despite these challenges, nucleic acid therapeutics might be powerful drugs for cancer treatment.

## Data Availability

The material supporting the conclusion of this review has been included within the article.

## Abbreviations

AML: Acute myeloid leukemia
ANXA1: Annexin A1
ASGPR: Asialoglycoprotein receptor
ASOs: Antisense oligonucleotides
asRNAs: Antisense RNAs
ATO: Arsenic trioxide
Bak1: Bcl-2 antagonist killer 1
CAR: Chimeric antigen receptor
Cas: CRISPR associated
CDK4/6: Cyclin-dependent kinase 4/6
ceRNAs: Competing endogenous RNAs
circRNA: Circular RNA
CRC: Colorectal cancer
CRISPR: Interspaced short palindromic repeats
CSC: Cancer stem cell
CTLA-4: Cytotoxic T-lymphocyte-associated protein 4
DCPS: Decapping enzyme scavenger
DHFR: Dihydrofolate reductase
DNA-PKcs: DNA-dependent protein kinase catalytic subunit
DOX: Doxorubicin
EMT: Epithelial–mesenchymal transition
gRNAs: Guide RNAs
HCC: Hepatocellular carcinoma
HOTAIR: HOX transcript antisense RNA
ICAM-2: Intercellular adhesion molecule-2
KRAS: Mutated Kirsten rat sarcoma
LAPC: Locally advanced pancreatic cancer
LNAs: Locked nucleic acids
lncRNAs: Long noncoding RNAs
LNPs: Lipid-based nanoparticles
MALAT1: Metastasis-associated lung adenocarcinoma transcript 1
miRNAs: MicroRNAs
MO: Morpholino oligonucleotides
MRP1: Multidrug resistance-associated protein 1
NATs: Natural antisense RNAs
ncRNAs: Noncoding RNAs
PBMCs: Peripheral blood mononuclear cells
PD1: Programmed cell death 1
PDL1: Programmed death-ligand 1
RISC: RNA-induced silencing complex
RNAi: RNA-mediated interference
saRNAs: Small activating RNAs
sgRNA: Single-guide RNA
shRNA: Short hairpin RNA
siRNAs: Small interfering RNAs
sno-lncRNAs: snoRNA-related lncRNAs
sRNAs: Small RNAs
TKIs: Tyrosine kinase inhibitors
TRAD: Telomerase-specific replication-competent adenovirus
tRNA: Transfer RNA
TS: Thymidylate synthase
XIST: X-inactive specific transcript
